# Investigation of PVT-Aware STT-MRAM Sensing Circuits for Low-VDD Scenario

**DOI:** 10.3390/mi12050551

**Published:** 2021-05-12

**Authors:** Zhongjian Bian, Xiaofeng Hong, Yanan Guo, Lirida Naviner, Wei Ge, Hao Cai

**Affiliations:** 1 National ASIC System Engineering Center, Southeast University, Nanjing 210096, China; 213173485@seu.edu.cn (Z.B.); hongxf@seu.edu.cn (X.H.); guoyn@seu.edu.cn (Y.G.); duiker@seu.edu.cn (W.G.); 2 Laboratoire Traitement et Communication de L’Information, Télécom Paris, 91120 Palaiseau, France; lirida.naviner@telecom-paristech.fr

**Keywords:** MRAM, sensing margin, yield, low TMR sensing, PVT variation, logic-in-memory

## Abstract

Spintronic based embedded magnetic random access memory (eMRAM) is becoming a foundry validated solution for the next-generation nonvolatile memory applications. The hybrid complementary metal-oxide-semiconductor (CMOS)/magnetic tunnel junction (MTJ) integration has been selected as a proper candidate for energy harvesting, area-constraint and energy-efficiency Internet of Things (IoT) systems-on-chips. Multi-VDD (low supply voltage) techniques were adopted to minimize energy dissipation in MRAM, at the cost of reduced writing/sensing speed and margin. Meanwhile, yield can be severely affected due to variations in process parameters. In this work, we conduct a thorough analysis of MRAM sensing margin and yield. We propose a current-mode sensing amplifier (CSA) named 1D high-sensing 1D margin, high 1D speed and 1D stability (HMSS-SA) with reconfigured reference path and pre-charge transistor. Process-voltage-temperature (PVT) aware analysis is performed based on an MTJ compact model and an industrial 28 nm CMOS technology, explicitly considering low-voltage (0.7 V), low tunneling magnetoresistance (TMR) (50%) and high temperature (85 °C) scenario as the worst sensing case. A case study takes a brief look at sensing circuits, which is applied to in-memory bit-wise computing. Simulation results indicate that the proposed high-sensing margin, high speed and stability sensing-sensing amplifier (HMSS-SA) achieves remarkable performance up to 2.5 GHz sensing frequency. At 0.65 V supply voltage, it can achieve 1 GHz operation frequency with only 0.3% failure rate.

## 1. Introduction

Perpendicular anisotropy-based magnetic tunnel junctions (p-MTJs) have been extensively studied to develop spin-transfer torque magnetic random access memories (STT-MRAMs) [1,2,3]. Hybrid MTJ/CMOS integration is developed with device scaling down to feature small dimension and low-power operations. STT-MRAM has been regarded as a potential candidate in the next-generation nonvolatile memories [4,5,6,7]. Compared to resistive random access memory (RRAM) and phase-change random access memory (PRAM), MRAM has a low read margin, but is suitable for high density integration and has high endurance, so the benefits can be greater if all design challenges are addressed to meet the design targets and achieve cost efficiency [8]. MTJ also shows CMOS compatibility thanks to the integration with Back End of Line (BEOL) process. Above merits enable MRAM replacement of SRAM/flash memory, especially for embedded systems and their applications.

The sensing amplifier (SA) or sensing circuit is an indispensable building block in spintronics-based circuits [4,5,6,7,9,10,11]. The latest sensing amplifier circuits for MRAM are detailed in [8]. low-VDD scenario, major design concerns of SA include sensing speed, margin and yield performance [12]. In general, MRAM sensing performance is dependent on hybrid process, voltage and temperature of surrounding environment, as well as aging degradation, which may suffer from read-disturbance and read-decision failure issues [6,7,9,10,11,13,14]. Redundancy and error-correcting code (ECC) techniques are normally used to solve the above reliability issues, which may deteriorate power-performance-area (PPA) metrics. Besides, scaling down of MTJ/CMOS device dimension gives rise to MRAM design challenges, mainly related to the insufficient sensing margin and increased sensing error rate.

Two SA modes were reported in previous work. Voltage-mode SA (VSA) is employed in previous small-$I_{cell}$ memory (non-volatile memories (NVMs) and low-voltage static random access memories (SRAMs) designs [12,15,16]. Although VSA benefits long BL developing time with bit-line (BL) and SA offset tolerance, long sensing latency becomes a critical issue, whereas current-mode SA (CSA) achieves improved sensing speed than VSA with reduced cell current [12,17,18,19]. And offset canceling technology for CSA is introduced in detail in [20,21,22].

Based on an industrial 28-nm CMOS process and MTJ compact model, this study investigates the performance of six CSAs, considering wide sensing supply voltage (VDD) and temperature range, as well as process fluctuation. We propose a low-voltage sensing circuits to enable low-power scenario operation of MRAM. Our contribution are summarized as follows:

- Six typical current-mode SAs are process-voltage-temperature (PVT) studied at 28-nm CMOS node. The analysis is based on a unitary transistor sizing rule to enable a fair comparison of sensing circuits.
- We propose a novel current-mode SA named 1D high-sensing 1D margin, high 1D speed and 1D stability (HMSS) SA, with dual reference configuration to enlarge sensing margin, the modified pre-charged pMOSFET to improve the sensing uniformity of logic ‘0’ and ‘1’.
- For low-VDD scenario, MRAM sensing variability, yield and failure should be emphasized, with the design trade-off of energy consumption and layout area.
- A modified spintronics-based logic-in-memory (LIM) scheme is proposed. The proposed HMSS-SA configures the high-sensing margin in-memory bit-wise computing with reduced failure probability.

The remainder of this paper is organized as follows. Previous current-mode SAs and our proposed HMSS-SA are discussed in Section 2. Section 3 performs the simulation and analyzes the sensing power-delay trade-off, process-temperature variations, sensing failure issues and low-$V_{dd}$ design boundary. In Section 4, a modified logic-in-memory scheme is implemented using HMSS-SA for in-memory bit-wise computing, and we provide conclusion in Section 5.

## 2. Preliminary

### 2.1. STT-MRAM Bit-Cell

The p-MTJs with MgO/CoFeB/heavy metal (e.g., Ta, Hf) structures bring a reasonable magnetoresistance ratio (TMR). Using a double MgO/CoFeB interface free layer and a single interface, the p-MTJs also possess a considerable thermal stability factor (Δ) and high switching current density [1,2]. A sufficient write current ($I_{c0}$) is required for changing between the parallel (P) and antiparallel (AP) MTJ states.

Typical flash-like MRAM bit-cell is configured with one MTJ connected in series with one access transistor as the 1T-1M structure. MTJ free layer is connected to the bit-line (BL) of memory array. Important building blocks eg., bit-cells, reference generators and sense amplifiers constitute MRAM sensing circuit. The bit-cell resistance along the BL is determined by P or AP state of MTJ. The sensing current is compared with its reference value to decide the logic ‘1’ or ‘0’. Table 1 lists the physical parameters of STT-MTJ used in this work. Several reliability issues impact MRAM bit-cell performance. The magnetic thermal noise demonstrates as an additional three-dimension magnetic field [23,24]. Besides, fabrication variability of MTJ diameter, thickness of each layer (MgO, free and fixed) and thermal stability cause performance uncertainties in bit-cell [25].

### 2.2. Sensing Circuits for STT-MRAM

**Figure 1:**
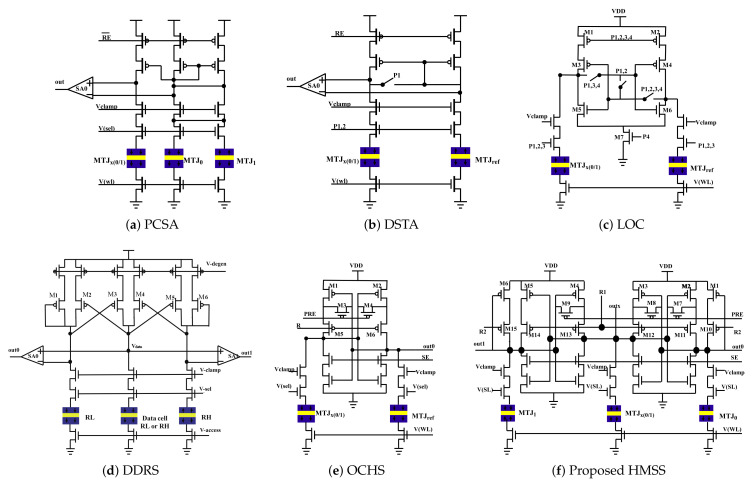
The schematics of sensing circuits. (**a**) Pre-charge SA (PCSA) [27]. (**b**) Double switches and transmission gate access transistor (DSTA) SA [28]. (**c**) Latch offset cancellation (LOC) SA [29]. (**d**) Dynamic dual-reference (DDRS) SA [30]. (**e**) Offset-compensated high-speed (OCHS) SA [31]. (**f**) High-sensing margin, high speed and stability (HMSS)SA.

Following current-mode sensing circuits for STT-MRAM are investigated in this work, the circuit schematics are demonstrated in Figure 1.

- Pre-charge sensing amplifier (PCSA) [27];
- Offset-compensated high-speed sensing (OCHS) [31];
- Dynamic dual-reference sensing (DDRS) [30];
- Latch offset cancellation sensing (LOC) [29];
- Double switches and transmission gate access transistor sensing (DSTA) [28];
- High-sensing margin, high speed and stability sensing (HMSS, proposed in this work).

The signals in the circuits listed are described below, “RE” is the enabling signal of the SA; “$V_{clamp}$” is the clamp voltage; “$V_{\left(sel\right)}$” is column select signal; “$V_{\left(wl\right)}$” is word line select signal and "PRE" is precharge signal. For other signals, please refer to the cited paper.

Table 2 lists the qualitative comparison of different sensing circuits, including the number of sensing path, the number of reference, P-channel metal–oxide–semiconductor (pMOS) load type and the reference scheme (CM for current-mean, RM for resistance-mean). Only transistor counts and minimum TMR are reported in the comparison, as different CMOS and MTJ process were used to realize previous sensing circuits [27,28,29,30,31,32]. Further quantitative analysis will be performed in Section 3. Low VDD (low sensing current) method is preferred to overcome unexpected spin inversion [33]. Although this method directly benefits low power consumption, the drawback is that sensing margin can be significantly limited, which causes sensing failure and yield degradation.

Conventionally, source degeneration and one-paired balanced reference scheme were used to improve process variation tolerance during MRAM sensing operation [27]. In [31], an offset-compensated high-speed sense amplifier (OCHS-SA) was implemented for high speed and high yield with offset voltage cancellation (see Figure 1e). It generates a voltage difference between $MTJ_{x}$ and $MTJ_{ref}$ path in pre-charge phase from $M_{2}$ and $M_{1}$ respectively. The next is the resistance change of $M_{1}$ and $M_{2}$ will amplify the voltage difference. Finally, the SE signal will open the latch to amplify the voltage difference to get an output voltage.

A dynamic dual-reference sensing (DDRS) scheme is proposed in [30]. DDRS can achieve a high sensing margin with the tradeoff such as slow speed, low yield and cannot solve the problem of offset voltage caused by PVT variation. The working principle of DDRS-SA is that a voltage difference will be generated between Data cell (if the data saved in Data cell is 0) and $R_{H}$ path. Then the voltage difference will be amplified through transistor P4 and P5. Finally, SA1 will amplify the voltage difference to generate the output. The sensing margin of OCHS-SA is lower than DDRS-SA and unbalance between read ‘0’ and read ‘1’.

In [29], the sensing circuit, latch sense amplifier and write driver are merged as a LOC-SA to reduce the voltage developing time, so that sensing latency can be significantly improved. The yield of LOC-SA is also enhanced through the offset cancellation scheme. In [28], double switch schemes with both foot-switch and head-switch have been used to overcome the invalid current problem (sensing dead zone). Last but not least, our proposed HMSS-SA demonstrates high sensing margin, fast speed, and high stability [32].

### 2.3. The Proposed HMSS Sensing Circuit

In order to achieve a high sensing margin in MRAM read operation, a novel sensing circuit implementation named HMSS-SA is proposed, as shown in Figure 1f.

- Pre-charge phase: PRE is set to low, $R_{1}$, $R_{2}$, $V_{clamp}$, $V_{SL}$, $V_{WL}$ are set to high. pMOS $M_{2}$, $M_{3}$ and $M_{4}$ are turned on for the pre-charge of the path of MTJx, MTJ0 and MTJ1 respectively. The output voltage of $out_{1}$ is higher, and the output voltage of $out_{0}$ is lower. Since the content of $MTJ_{x}$ is 1(0), the output voltage of $out_{x}$ is the same as that of $out_{1}$ ($out_{0}$).
- Sensing phase: PRE is set to high, $R_{1}$ and $R_{2}$ are set to low, and the voltage obtained by pre-charge is sensed and amplified. Since $out_{x}$ and $out_{1}$ ($out_{0}$) have the same voltage, the voltage of $out_{x}$ and $out_{1}$ ($out_{0}$) will be changed from the change between pMOS $M_{3}$ ($M_{4}$) and $M_{2}$ ($M_{5}$).It reaches the state where the $out_{x}$ output voltage is high (low) and $out_{1}$ and $out_{0}$ output voltage are low (high).
- Amplified phase: $V_{clamp}$, $V_{SL}$, $V_{WL}$ are set to low, SE is set to high, and the voltage of $out_{x}$ is rapidly increased (decreased) to the standard high (low) voltage by the influence of double latch. $out_{1}$ and $out_{0}$ are decreased (increased) to the standard low (high) voltage.

**Figure 2:**
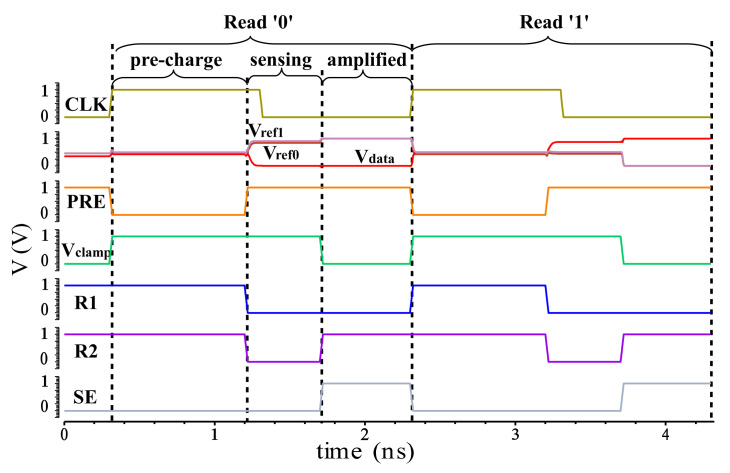
Simulated waveform of proposed HMSS sensing circuit.

Figure 2 illustrates the simulated waveform. The proposed HMSS-SA introduces $MTJ_{1}$ and $MTJ_{0}$ as double references to enlarge the sensing margin. The principle is that when the storage content of $MTJ_{x}$ is ‘0’ (see Figure 1f), the primary reference object is $MTJ_{1}$. When the storage content of $MTJ_{x}$ is ‘1’, the primary reference object is $MTJ_{0}$. Therefore regardless of the value stored in $MTJ_{x}$, the sensing margin of the circuit is always the voltage difference between the $MTJ_{1}$ and $MTJ_{0}$ paths. Since the circuit uses a double reference, the current in the $MTJ_{x}$ path is approximately twice the traditional signal reference sense amplifier. Therefore, in order to match the current in the $MTJ_{x}$, a dual pMOS method is adopted in both the $MTJ_{1}$ and the $MTJ_{0}$ path. During pre-charging, pMOS $M_{10-15}$ are in the off state, and $M_{7-9}$ are in the on state. At this time, the power supply pre-charges the $MTJ_{x}$, $MTJ_{0}$ and $MTJ_{1}$ paths through $M_{2-4}$ respectively. Selecting $M_{2}$ instead of $M_{5}$ as the pre-charge pMOS for $MTJ_{x}$ can greatly reduce the uniformity of reading ‘0’ and reading ‘1’. During the sensing phase, pMOS $M_{10-15}$ are in the on state, and $M_{7-9}$ is in the off state, so that the voltage difference between $out_{x}$ and $out_{1(0)}$ can be amplified. When the amplifier phase is reached, the three paths of $MTJ_{x}$, $MTJ_{0}$ and $MTJ_{1}$ are turned off, and $M_{10}$ and $M_{15}$ are in the off state. At this time, the double SA further amplifies the voltage difference between $out_{x}$ and $out_{1(0)}$.

Table 2 summarizes and compares the recently published sensing circuits according their performance in [27,28,29,30,31,32]. In next sections, the above mentioned sensing circuits will be evaluated with 28-nm CMOS technology.

### 2.4. Logic-in-Mram Application

The combination of MRAM and logical computing is a highly energy efficient approach. Since stored data has been already memorized into MTJ devices in the proposed circuits, the supply voltage can be immediately cut off without data transmission into external nonvolatile storage devices when the circuit changes to a standby mode. This property achieves great reduction of power dissipation [34,35,36].

## 3. PVT-Aware Analysis of Low-VDD MRAM Sensing Operation

### 3.1. Sensing Margin Estimation

Sensing margin (SM) = $|V_{REF}-V_{DATA}|$ increases as $|R_{REF}-R_{DATA}|$ increases. In a dual reference SA, when the data is ‘1’ (‘0’), the actual reference is $MTJ_{0}$ ($MTJ_{1}$). Comparing with the average resistance SA, $|R_{REF}-R_{DATA}|$ is two-fold increased, so that the sensing margin can be greatly enlarged.

Assume that σ is the PVT induced maximum voltage deviation produced by the load transistor $V_{TH}$ changing to the output (σ is the absolute value of the maximum deviation). The SM without the variation of the load pMOS transistor $V_{TH}$ is referred to as the ideal SM value. Equations (1) and (2) describe the sensing margin and σ when reading ‘0’ and reading ‘1’.

When reading logic ‘0’, $SM_{min.(w/o)}$ in Equation (1) describes the minimum value of SM without cross-precharge, such as PCSA and LOC, with a 2σ deviations from the nominal sensing margin. When designing with partially offset cancellation, such as OCHS and HMSS, $SM_{min.}$(the minimum value of SM) is with a deviation of σ from the nominal margin. Equation (2) explains the condition when reading logic ‘1’ *w/o* and with cross-precharge.

(1) $$\begin{array}{cc} SM_{min.(w/o)}=\left|V_{REF}-\sigma -\left(V_{DATA}+\sigma \right)\right|=V_{REF}-V_{DATA}-2\sigma \\ SM_{min.}=\left|V_{REF}-\left(V_{DATA}+\sigma \right)\right|=V_{REF}-V_{DATA}-\sigma & \\ \mathrm{For}\;\mathrm{MTJ}\;P\;\mathrm{state}\;\mathrm{sensing} \end{array}$$

(2) $$\begin{array}{c} SM_{min.(w/o)}=\left|V_{REF}+\sigma -\left(V_{DATA}-\sigma \right)\right|=V_{DATA}-V_{REF}-2\sigma \\ SM_{min.}=\left|V_{REF}-\left(V_{DATA}-\sigma \right)\right|=V_{DATA}-V_{REF}-\sigma \\ \mathrm{For}\;\mathrm{MTJ}\;\mathrm{AP}\;\mathrm{state}\;\mathrm{sensing} \end{array}$$

### 3.2. Energy-Delay Performance Evaluation

The analysis is executed with an experimental validated p-MTJ compact model to investigate the performance of sensing circuits [37,38]. 200 nm/30 nm width/length transistor dimension is used to design sensing circuits based on a sweep analysis for performance optimization. Regarding the process variations, the mean and standard deviation of parameters are estimated through Monte Carlo (MC) simulations. The sensing failure probability is analyzed under global process variation and local mismatch of 28-nm transistor and 40-nm-diameter STT-MTJ. The evaluations are performed in Cadence analog design environment with 1000 runs MC analysis. 1-sigma CMOS transistor variability is considered, whereas the Gaussian distribution is realized in STT-MTJ at the range 0.9 to 1.1.

**Figure 3:**
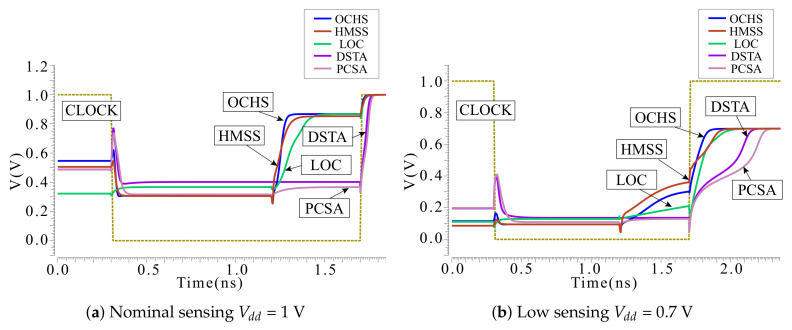
Sensing antiparallel (AP) as an example, simulated sensing operation waveform of OCHS, HMSS, LOC, DSTA and PCSA designed with 28-nm complementary metal-oxide-semiconductor (CMOS) with (**a**) nominal sensing $V_{dd}$ = 1 V and (**b**) low sensing $V_{dd}$ = 0.7 V. Pre-charge, sensing and amplified phases are sequentially demonstrated. The dotted line represents the sensing clock signal. DDRS waveform is separately demonstrated in Figure 4.

**Figure 4:**
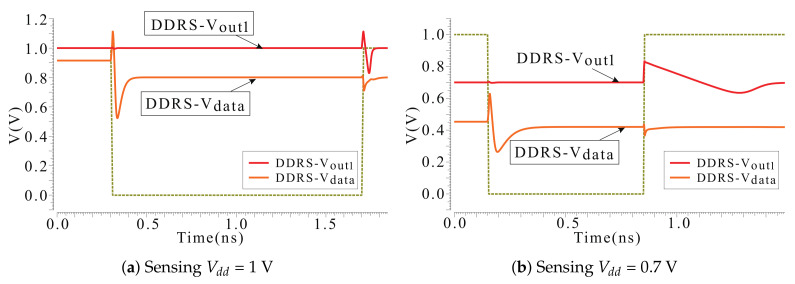
Sensing AP as an example, the sensing operation waveform of DDRS (see Figure 1d). As both sensing and amplify phase need to obtain the voltage changes between $V_{data}$ and out1, the DDRS waveform is separately demonstrated with (**a**) sensing $V_{dd}$ = 1 V and (**b**) sensing $V_{dd}$ = 0.7 V.

Figure 3 and Figure 4 are the waveform depicting the transient behavior of each circuit. Sensing AP as an example, the operation can be divided into two phases, one is the sensing phase before clock rising edge, the other is the amplify phase after the clock rising edge. Since the sensing phase of the DDRS and the amplify phase need to read the voltage changes between $V_{data}$ and out1, the DDRS waveform is separately shown here from the other waveforms. The clamp transistor that uses the $V_{clamp}$ as the gate voltage ensures that the voltage and current on the bit line within a certain range which will not change the state of the MTJs. Therefore, when the power supply voltage is reduced, $V_{clamp}$ should be reconfigured so as to obtain a higher read yield without changing the state of the MTJ. According to sensing methods, the sensing latency with different $V_{dd}$ can be obtained under the condition of adjusting the $V_{clamp}$.

Figure 3 and Figure 4 illustrate the sensing operation waveform, including pre-charge, sensing and amplified phases. The latency from beginning to stable of each phase is evaluated and accumulated, with the delay of the amplifier stage as the the total sensing latency.

**Figure 5:**
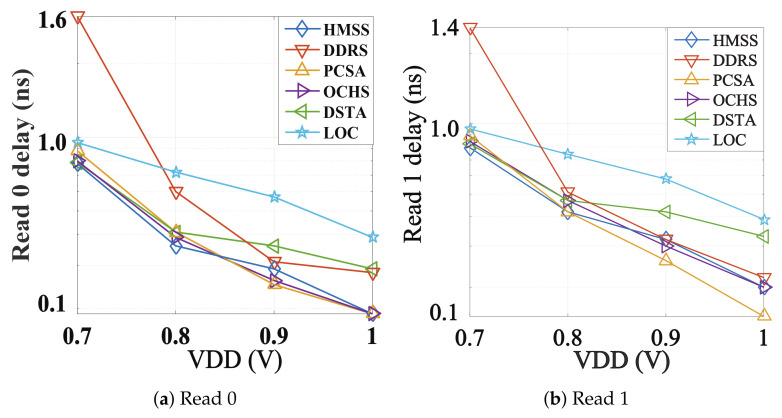
The comparison of sensing latency with (**a**) reading 0 and (**b**) reading 1.

Figure 5 compares sensing latency performance. LOC is with the worst sensing latency due to the multi-phases (equalizing, voltage developing, comparison and latching) sensing mechanism. In general, the delay of DDRS at 0.7 V is the largest, but at 0.8 V to 1 V the delay of LOC is the largest. At the same time, as the voltage increases, the delay of DSTA falls slower than others. Thus, DSTA latency is slightly higher than other SAs except LOC in the range from 0.8 V to 1 V. PCSA, OCHS and the proposed HMSS maintain an enhanced sensing speed over the 0.7 V to 1 V voltage range. The reason why the delay of DDRS increases so much at low voltage is that the voltage difference (obtained through reading circuit) is the gate voltage of the N-Metal-Oxide-Semiconductor (NMOS) pair to control the amplifier. The discharge current is immediately decreased when ultra-low $V_{G}$ is biased. The latency is simultaneously increased.

**Figure 6:**
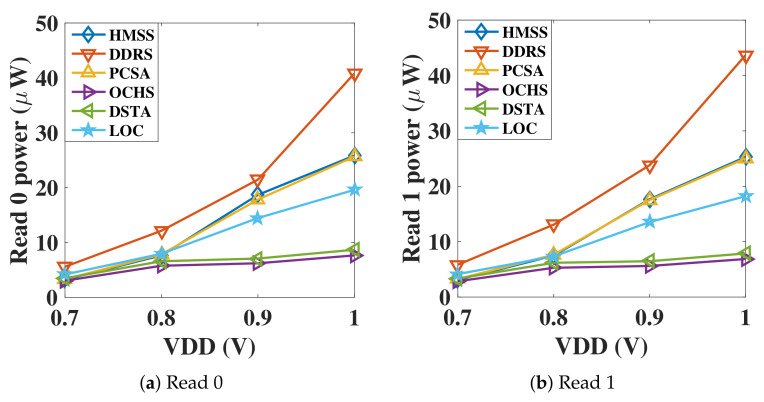
The dynamic sensing power consumption of different sensing circuits with (**a**) reading 0 and (**b**) reading 1. Due to the triple paths structure, the dynamic power dissipation of our proposed HMSS-SA is greater than OCHS but less than DDRS.

The dynamic power consumption is evaluated through averaging the power dissipation in several operating phases. Figure 6 shows the comparison of the dynamic power of ‘P’ state and ‘AP’ state sensing, at the voltage range of 0.7 V to 1 V. Notice that DDRS has the largest dynamic power and OCHS has the lowest dynamic power. The dynamic power consumption of LOC, PCSA and the proposed HMSS is in the middle level, whereas the DSTA is slightly higher than the OCHS. Due to the triple paths from $V_{DD}$ to ground, DDRS, PCSA and the proposed HMSS achieve the largest dynamic power, whereas the proposed HMSS is designed with the lowest power consumption compared with DDRS and PCSA.

**Figure 7:**
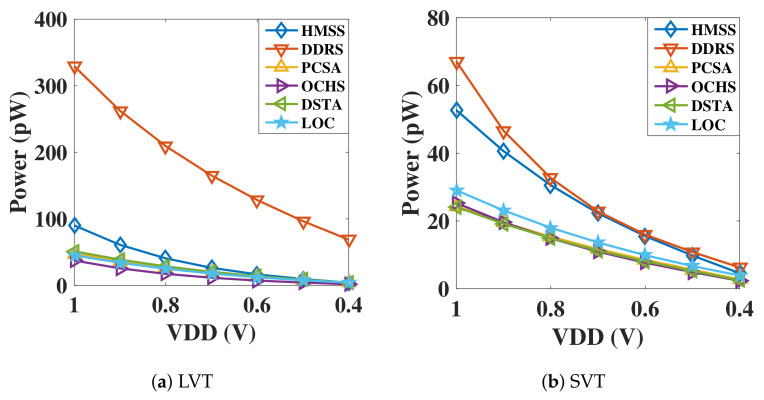
Static power consumption versus $V_{dd}$ scaling down in sensing circuits with (**a**) low VT transistor and (**b**) regular VT transistor.

Figure 7 shows the static power of six SAs over a wide voltage range. Notice that whether using standard $V_{TH}$ (SVT) or low $V_{TH}$ (LVT) transistor, DDRS-SA is with the highest static power dissipation, whereas OCHS-SA achieves the lowest leakage cost. In addition, the static power of the proposed HMSS is at a high level, and the static power of the remaining three sense amplifiers is at an intermediate level. According to the analysis of the circuit, it can be found that the more the pMOS connect to VDD and the number of paths to the ground, the larger the static power of the circuit is. When operating at low-VDD region, the static power performance of SAs is not obvious except DDRS-SA designed with LVT transistor.

### 3.3. Low-VDD Sensing

**Figure 8:**
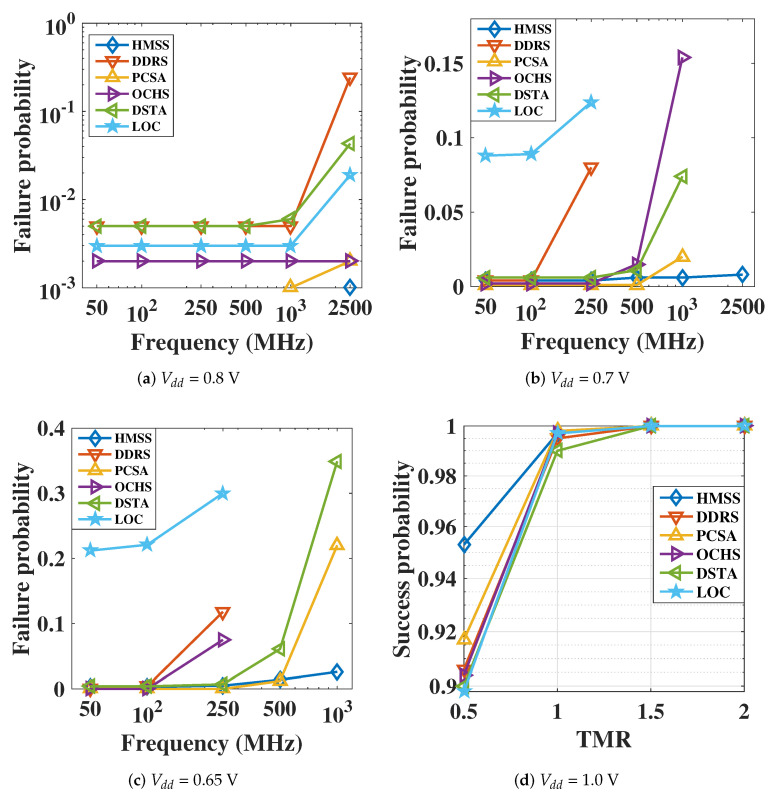
(**a**–**c**) Sensing failure probability versus sensing frequency at different $V_{dd}$ node. TMR = 100%. (**d**) Successful sensing probability versus MTJ TMR at 1V $V_{dd}$. HMSS achieves an improved sensing rate even that the TMR was as low as 50%.

Using the optimized $V_{clamp}$ and transistor dimension sizing, low Low-VDD operation can be realized in different sensing circuits. The $V_{clamp}$ is configured to reach the maximum that satisfies the unwritten condition at low supply voltages. Figure 8a–c illustrates the sensing failure probability versus frequency at different $V_{dd}$ nodes (TMR = 100%). The proposed HMSS shows the up to 2.5 GHz high frequency performance with nominal $V_{dd}$. At 0.65 V $V_{dd}$ node, it can achieve 1 GHz operation frequency with 0.3% failure rate.

Figure 8d shows the successful sensing probability versus TMR equals to 50%, 100%, 150% and 200%, under 1 V $V_{dd}$. Notice that when TMR is greater than 150%, the successful sensing rate reaches 100% in the 1000 runs MC analysis. Compared with other SAs (with performance optimization), the optimized HMSS has the enhanced sensing probability (4–5.5% improvement) even with TMR at 50%.

### 3.4. Temperature-Aware Sensing

**Figure 9:**
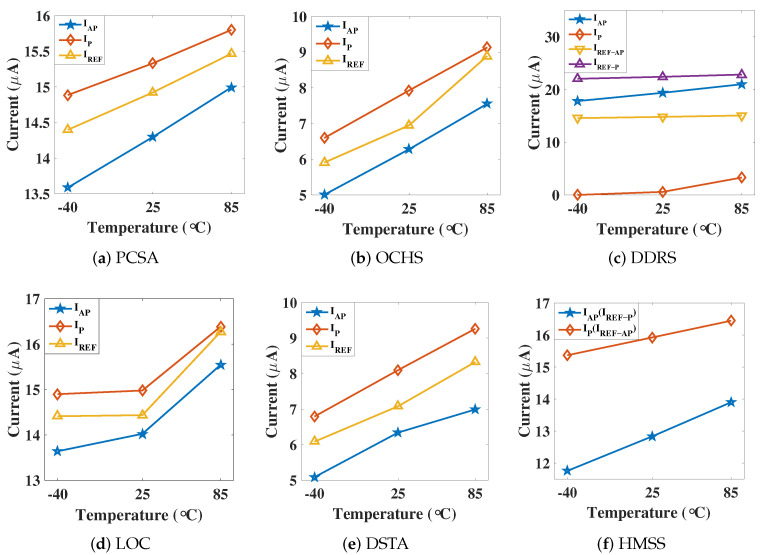
Sensing current versus temperature analysis in different sensing circuits: (**a**) PCSA, (**b**) OCHS, (**c**) DDRS, (**d**) LOC, (**e**) DSTA, (**f**) HMSS. The sensing margin at low temperature is greater than high temperature. Note that the sensing current in different circuits are lower than MTJ critical current ($I_{c0}$ = 50 µA).

In order to reduce sensing failure probability, an ideal reference is preferred to locate in the middle of the read window ($I_{p}$ & $I_{ap}$ mean). An evaluation of sensing current versus operation temperature is depicted in Figure 9. $I_{AP}$ is the current of the data path when reading logic ‘1’, whereas $I_{P}$ is for logic ‘0’. $I_{REF}$ is the current of reference path. As shown in Figure 9c,f, $I_{REF-AP}$ ($I_{REF-P}$) is the average current of the two reference paths when reading logic ‘1’ (logic ‘0’) in the dual reference scheme. During the reading process, the DDRS-SA is implemented without clear demarcation point during the precharge phase and the differential voltage development phase, resulting in a large data path current when reading ‘1’ in the steady state and a relatively small current when reading ‘0’. Meanwhile, as DDRS is designed with dual reference scheme, the current of the ‘AP’ reference path and the ‘P’ path is also different.

The current difference between the AP and *P* reference path is around 2 µA to 6 µA, the current of the reference path is taken as the average current of the AP and the P reference path. The $I_{REF}$ in Figure 9a,e are approximately located in the middle of $I_{P}$ and $I_{AP}$ (sensing window), which shows robust performance in low and high temperature. The $I_{REF}$ in OCHS is close to $I_{P}$ at high temperature which exhibiting the sensitive to temperature changes of these two SAs. The relationship of current and temperature of the proposed HMSS is depicted in Figure 9f. When data is AP, the reference is with P path, so the $I_{REF-AP}$ is changed to $I_{P}$. When data is P, the reference is AP path, so $I_{REF-P}$ = $I_{AP}$. We notice that the proposed HMSS has stable performance in temperature changes.

### 3.5. Discussion

Table 3 lists the simulation results of different sensing circuits. The proposed HMSS demonstrates a better sensing margin, faster speed and higher stability. Compared with the other dual-reference sensing amplifier (DDRS, theoretically with the same sensing margin), it demonstrates an improved sensing speed and less variability induced failure. The success sensing rate is higher than that of the other dual reference SAs.

Compared to the OCHS-SA with the lowest dynamic power consumption, the sensing margin of HMSS is about twice of OCHS, which means that in the case of the immature MTJ fabrication process, it contributes much more to the stability of MTJ reading. Secondly, with 0.7 V $V_{dd}$ and the same configuration of transistor, the success rate reading ‘0’ and reading ‘1’ is about 95.4% and 96.4%, and the success rate of OCHS sensing amplifier for reading ‘1’ is only 91.8%. There is also a serious disparity when reading ‘0’.

Nominal/high VDD can effectively guarantee the sensing margin and speed. However, high sensing VDD may induce the read disturbance. For current sensing scheme, clamp transistor with $V_{clamp}$ must be carefully designed. For low-VDD implementation in MRAM sensing, the trade-off of yield, speed, power and area are sequentially considered and optimized in this work. In fact, no matter MRAM is implemented with low or high-VDD, high successful sensing probability must be guaranteed to alleviate the workload of ECC blocks.

We also notice that some design details must be emphasized, e.g., reference scheme. If applying the local reference scheme to previous SAs to track bit-cell variations (as in the proposed SA), the power consumption of previous SAs will be larger than this work. In this work, the SA implementation has not been hierarchically related to the higher system/chip level. Considering the entire MRAM macro, additional power consumption and layout area is a small portion when comparing with error-coding correction blocks and redundancy blocks.

## 4. Low-VDD Sensing: A Case Study in Logic-in-Memory

### 4.1. The Modified Logic-in-Memory

A promising candidate to achieve energy-efficient spintronic circuit design is to simultaneously use MTJs for storage units and logic operation/computation. Spintronics-based bit-wise processing-in-memory (PIM), computing-in-memory (CIM), logic-in-memory (LIM) maily rely on CMOS circuit-level implementation for logic operation, which can achieve massive parallelism, high bandwidth and high density while minimizing power and cost [9,39,40,41]. Typical spintronics-based Pinotubo [39], STT-CIM [40], and NV-LIM [9] require SA modification, as well as additional reference circuits to support logic operations. A true Spintronics PIM semantic is proposed within a RAM array as distinguished from previous CMOS-based solutions, which is referred as computational RAM (CRAM) [41]. Among these schemes, NV-LIM is a prototype validated method using additional pass-transistor-logic network within MTJ nonvolatile data sensing paths [9].

**Figure 10:**
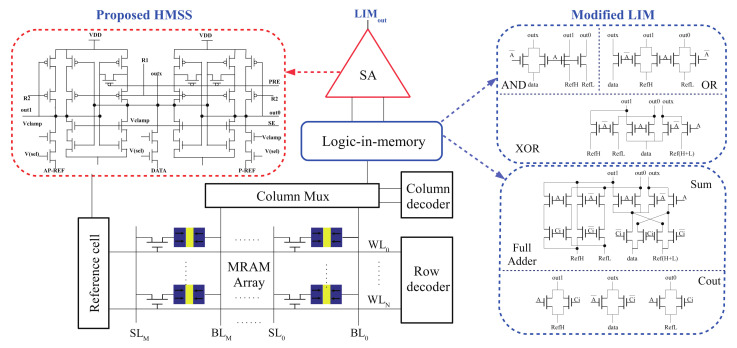
HMSS-SA with improved sensing margin applied to the modified logic-in-memory scenario.

Regardless of in memory computing schemes, the SA circuit is an indispensable building block in spintronics-based circuits. In order to further demonstrate HMSS-SA performance in LIM scenario, a modified NV-LIM block diagram is demonstrated in Figure 10, including Bit-wise operations AND, OR, XOR as well as the full adder.

PCSA can effectively perform OR operations and AND operations, but cannot perform a correct XOR operation, as the reference path and the data path need to be exchanged when performing XOR. PCSA uses the average current of the two reference paths taken by the CM reference, the reference path and the data path cannot be normally exchanged.

OCHS, DSTA and LOC can be directly combined with the LIM as described in Figure 10. The principles of OR, AND and XOR are implemented by LIM as follows (B is the data stored in MRAM, A is the data that has been read and applied to the control transistor): (1) The implementation of the OR operation, when A = ‘0’, B is normally read as the operation result, when A = ‘1’, the data path portion of the LIM is turned off, the output result is always ‘1’; (2) The implementation of the AND operation, when A = ‘1’, B is normally read as the operation result, when A = ‘0’, the reference path portion of the LIM is turned off, the output result is always ‘1’; (3) Implementation of XOR operation, when A = ‘0’, B is normally read as the operation result, when A = ‘1’, the data path of LIM is exchanged with the reference path, and the data is read as the operation result.

DDRS and HMSS are implemented with the dual-reference scheme so that a modified LIM is required, which comes from: (1) single MTJ is used to store one bit of data, whereas two MTJs are used to store one bit of data in previous literature. (2) The SA in the LIM is with dual-paths, whereas DDRS and HMSS are implemented with triple-path in this work.

### 4.2. Failure Probability of Modified LIM

Table 4 compares the bit-wise computation failure rate when using different sensing circuits in the modified LIM structure under 0.7 V $V_{dd}$. Notice that the failure probability in the modified LIM is lower than sensing circuits. The reason is that when the data path portion (in OR operation) and the reference path portion (in AND operation) are turned off, the output result of data path and reference path is always logic ‘1’ so that 100% sensing probability can be guaranteed.

## 5. Conclusions

In this work, previous MRAM sensing circuits were investigated using 28-nm CMOS technology with process-voltage-temperature aware considerations. A novel sensing circuit named HMSS was proposed for low-VDD high yield MRAM design. The proposed circuit uses the current model, dual reference scheme as well as modified pre-charged pMOSFET to enhance the sensing margin. The simulation results show that HMSS achieved high sensing speed at 1 V nominal $V_{dd}$, and low failure probability (0.4% with TMR = 100%) at 0.7 V low $V_{dd}$. Process variations, wide temperature range and $V_{dd}$ scaling were investigated for sensing operation with high reliability. Compared with previous works, HMSS achieved an improved successful sensing rate even the TMR was as low as 50%. A modified logic-in-memory circuit was implemented with reduced sensing probability. The presented results give useful insights in the 28-nm node MRAM sensing circuit, and provide design guidelines for logic-in-memory spintronics circuits and architectures. 

## Figures and Tables

**Table 1:** Physical properties and design parameters of spin-transfer torque-magnetic tunnel junction (STT-MTJ) and magnetic random access memory (MRAM) bit-cell [1,26].

Parameter	Description	STT-MTJ
TMR	tunneling magnetoresistance (TMR) ratio	50–200%
$T_{ox}$	magnetic tunnel junction (MTJ) oxide thickness	0.85 nm
$T_{sl}$	MTJ free layer thickness	1.3 nm
Area	MTJ layout surface	40 nm × 40 nm
$R_{P}$/$R_{AP}$	MTJ resistance	6.2 kΩ/18.6 kΩ
α	Damping factor	0.027
Δ	Thermal stability	72
$I_{c0}$	Write critical current	50 µA
*T*	Ambient temperature	243 K, 300 K, 358 K
$V_{dd-sense}$	Sensing supply voltage	0.6–1 V
W/L	Transistor dimension	W = 200 nm, L = 30 nm

**Table 2:** Qualitative comparison of different sensing circuits.

	IEEE Transactions on Very Large Scale Integration (TVLSI)’12	TVLSI’14	TCAS-1’15	TCAS-1’17	TVLSI’18	This Work
Name/Abbreviation	Pre-charge sensing amplifier (PCSA)	Double switches and transmission gate access transistor sensing (DSTA)	Latch offset cancellation (LOC)	Dynamic dual-reference sensing (DDRS)	Offset-compensated high-speed sensing (OCHS)	High-sensing margin, high speed and stability sensing (HMSS)
Technology node (reported) *	65 nm	65 nm	45 nm	40 nm	65 nm	28 nm
Number of path	3	2	2	3	2	3
Number of reference	single	single	single	dual	single	dual
Reference scheme **	CM	RM	RM	CM + RM	RM	CM + RM
Number of transistor	21 T	22 T	19 T	39 T	16 T	33 T
Suggest $V_{dd}$	1.1 V	1.1 V	1 V	1 V	0.75 V	0.7 V
Minimum TMR (reported)	150%	N/A	150%	50%	100%	50%
Voltage of pMOS load	fixed	fixed	Varied	Varied	Varied	Varied
Width of pMOS load	3.3 µm	N/A	>1 µm	0.24 µm	0.6 µm	0.2 µm

* Previous sensing circuits will be further analyzed with 28-nm complementary metal-oxide-semiconductor (CMOS) technology in Section 3. ** The type of reference scheme: CM for current-mean scheme, RM for resistance-mean scheme.

**Table 3:** Summary of Low-VDD sensing circuit performance ${}^{*}$.

		T-VLSI’12	T-VLSI’14	TCAS-1’15	TCAS-1’17	T-VLSI’18	Proposed
Name/Abbreviation		PCSA	DSTA	LOC	DDRS	OCHS	HMSS
Dynamic power (µW)		25.36	8.29	18.92	42.26	7.263	25.57
Leakage power@$V_{dd}$ = 1 V (*p*W)		24.35	24.16	29.07	67.14	25.31	52.75
Sensing latency ** (ns)		0.19	0.33	0.39	0.28	0.2	0.2
Sensing current (µA)		15.3	7.95	19.9	15	8.1	15.92
Minimum sensing $V_{dd}$ realized in 28 nm node (V)		0.65	0.65	0.7	0.55	0.65	0.65
Failure probability (%)	TMR = 100% @$V_{dd}$ = 1 V	0.2	1	0.3	0.5	0.3	0.3
	TMR = 100% @$V_{dd}$ = 0.7 V	0.4	1.1	1.3	0.4	0.2	0.4
	TMR = 50% @$V_{dd}$ = 1 V	8.3	10	10.2	10.1	9.4	4.7
	TMR = 50% @$V_{dd}$ = 0.7 V	8.6	10.3	14.8	10.7	9.5	5.2
Maximum sensing frequency (MHz)	$V_{dd}$ = 0.7 V, Yield > 90%	1000	1000	100	250	500	2500
	$V_{dd}$ = 0.7 V, Yield > 99%	500	250	N/A	100	250	2500
Sensing current margin *** (µA)	@−40${}^{\circ }$	1.299	1.706	1.257	N/A	1.592	3.611
	@25 ${}^{\circ }C$	1.034	1.757	0.954	N/A	1.65	3.088
	@85 ${}^{\circ }C$ (worst case)	0.807	2.271	0.841	N/A	1.58	2.547

* All sensing circuits are implemented with 28-nm CMOS technology. 200 nm/30 nm transistor dimension are mainly used. Performance optimization is executed by adjusting the dimension of several transistors. ** Sensing latency is the time from the beginning of the sensing phase to the stable of output voltage. *** Current difference between $I_{AP}$ and $I_{P}$ ($I_{AP}$/$I_{P}$, current flowing through $R_{AP}$/$R_{P}$). Due to the feedback path in DDRS, current margin is not recorded.

**Table 4:** The failure probability of LIM bit-wise operation with low TMR=50%.

TMR = 50%	PCSA	DSTA	LOC	DDRS	OCHS	HMSS
	**$V_{\mathrm{dd}}=1.0$ V**					
OR/AND	4.1%	5.0%	5.1%	4.7%	4.6%	2.3%
XOR	N/A	10.1%	10.2%	9.9%	9.35%	6.85%
	**$V_{\mathrm{dd}}=0.7$ V**					
OR/AND	4.3%	5.15%	7.4%	5.35%	4.75%	2.6%
XOR	N/A	10.3%	14.8%	10.1%	9.5%	6.95%
